# Gene Expression Pattern Associated with Cytoskeletal Remodeling in Lipid-Loaded Human Vascular Smooth Muscle Cells: Crosstalk Between C3 Complement and the Focal Adhesion Protein Paxillin

**DOI:** 10.3390/cells14161245

**Published:** 2025-08-12

**Authors:** Maisa Garcia-Arguinzonis, Rafael Escate, Roberta Lugano, Esther Peña, Maria Borrell-Pages, Lina Badimon, Teresa Padro

**Affiliations:** 1 Institut Recerca Sant Pau (IR-Sant Pau), 08041 Barcelona, Spain; mgarciaar@santpau.cat (M.G.-A.); rescate@santpau.cat (R.E.); epena@santpau.cat (E.P.); mborrellpa@santpau.cat (M.B.-P.); lbadimon@ficsi.org (L.B.); 2 Centro de Investigación Biomédica en Red Cardiovascular (CIBERCV), Instituto de Salud Carlos III, 28029 Madrid, Spain; 3 Candiolo Cancer Institute, Fondazione del Piemonte per l’Oncologia (FPO-IRCCS), Candiolo, 10060 Torino, Italy; roberta.lugano@ircc.it; 4 Medical School, Universitat de Vic–UCC, Cardiovascular Research Foundation for Health Prevention and Innovation (FICSI), 08017 Barcelona, Spain

**Keywords:** cell migration, cell adhesion, cytoskeleton, actin organization, gene expression

## Abstract

Mechanical and contractile forces in the vascular wall regulate smooth muscle cell migration. We previously demonstrated the presence of C3 complement products in atherosclerotic lesions of human aortas and showed that that C3-derived fragments promote key cellular processes, such as actin cytoskeleton organization and cell migration, in lipid-loaded human vascular smooth muscle cells (hVSMCs). In the present study, we aimed to investigate gene expression profiles related to cytoskeletal remodeling and cell adhesion in migrating hVSMCs with a particular focus on modulatory effect of the C3 complement pathway on these processes. We analyzed gene expression in migrating and non-migrating hVSMCs using real-time PCR and in silico network analysis. Additionally, we investigated cytoskeletal remodeling through Western blotting and confocal microscopy. PCR profiling revealed 30 genes with significantly altered expression in migrating hVSMCs compared to non-migrating control cells. In silico analysis identified six of these genes—PXN, AKT1, RHOA, VCL, CTNNB1, and FN1—as being associated with cytoskeletal remodeling and focal adhesion, with PXN occupying a central position in the interaction network. PXN expression was reduced at both the transcript and protein levels and showed altered subcellular localization in migrating lipid-loaded hVSMCs. Protein–protein interaction analysis using STRING predicted an association between PXN and the integrin complex αMβ2 (comprising ITGAM (CD11b) and ITGB2 (CD18)), which functions as receptors for the iC3b complement fragment. Confocal imaging of cell adhesion structures revealed that lipid-loaded hVSMCs stimulated with iC3b displayed a more diffuse PXN distribution and significantly increased PXN–F-actin colocalization in active cytoplasmic regions compared to lipid-loaded control cells. PXN–F-actin colocalization increased from 1.26% to 19.68%. Subcellular fractionation further confirmed enhanced PXN enrichment in the membrane fraction, with no significant changes observed in the cytosolic or cytoskeletal compartments. In conclusion, iC3b-mediated molecular signaling in lipid-loaded hVSMCs alters PXN distribution and enhances cytoskeletal remodeling, revealing novel molecular interactions in vascular remodeling and the progression of atherosclerotic lesions.

## 1. Introduction

Vascular smooth muscle cell (VSMC) proliferation and migration play a key role in vascular remodeling in various cardiovascular diseases, including atherosclerosis and restenosis following vascular intervention [1,2,3,4]. Cell migration is a finely regulated dynamic process that requires coordinated interactions between intracellular proteins and extracellular matrix (ECM) components. These interactions lead to the formation of lamellipodia and new focal adhesions at the leading edge of migrating cells, strengthening their attachment to the ECM to facilitate reorganization of the actin cytoskeleton [5,6].

Previous studies from our group have shown that aggregated and proteoglycan-modified lipids internalized by the LRP1 receptor induce functional and structural changes in VSMC [7]. Internalized LDL cause modulation of HSP27 localization, with effects on actin polymerization and cytoskeleton dynamics [8], impairment of cell migration [9,10], and a decrease and delocalization of profibrinolytic receptors that result in a general impairment of cytoskeleton dynamics and adhesion capacity, affecting cell phenotype and function [10].

We have previously reported the abundance of C3 complement products in atherosclerotic lesions of human aortas and demonstrated their capacity to stimulate actin cytoskeleton organization, cell migration, and cell–matrix adhesion in human lipid–loaded VSMCs [11]. In this context, knockout of C3 expression (C3^−^/^−^) in murine models of atherosclerosis (Ldlr^−^/^−^ or ApoE^−^/^−^ Ldlr^−^/^−^ background) has been associated with reduced VSMC and collagen content in atherosclerotic lesions—hallmarks of plaque vulnerability [12,13]. Together, these findings provided the functional rationale for the present work, which builds on those results by examining the associated transcriptional changes and molecular interaction networks involved in cytoskeletal remodeling and adhesion-related signaling.

Adaptor and scaffold proteins involved in cell cytoskeleton organization, such as those of the paxillin family, localize at the cell–ECM interface within focal adhesion sites [14]. Paxillin acts downstream of the RhoA signaling pathway and regulates actin stress fiber formation and cell migration [15]. Coordinated turnover of focal adhesion function is essential for cellular migration. Understanding the mechanisms that regulate this turnover is becoming increasingly important, particularly to explain cell recruitment during inflammation, tissue damage, or healing. Recent research has established links between cytoskeleton remodeling, focal adhesion, and LDL in VSMC migration, particularly through mechanisms involving Arp2/3-mediated mitochondrial dynamics [16].

Despite significant progress in understanding VSMC function, the genetic interactions underlying cytoskeletal remodeling and focal adhesion during cell migration remain poorly defined, particularly in the context of modulation by the C3 complement system.

Here, we applied a targeted transcriptomic and network analysis approach to characterize gene expression changes linked to cytoskeletal remodeling in lipid-loaded human vascular smooth muscle cells (hVSMCs) and to assess how lipid loading and C3 complement activity (iC3b) modulate adhesion-related molecular pathways within a hypothesis-generating, pathway-focused framework.

## 2. Materials and Methods

### 2.1. hVSMC Culture and LDL Preparation

Primary hVSMCs (purchased from Cell Applications, Inc, San Diego, CA, USA) were cultured in M199 medium containing 20% FBS and used between passage four and seven, as previously described [11]. The experiments were performed in subconfluent monolayers, cultured to reach approximately 90% confluence over 16 h.

Human LDLs (density 1.019–1.063 g/mL) were obtained by sequential ultracentrifugation [17] from a pooled sample of more than 100 anonymized residual serum specimens from normo-cholesterolemic donors, provided by the Barcelona Blood and Tissue Bank. LDL protein levels were quantified by the bicinchoninic acid (BCA) assay (Pierce) and agarose gel electrophoresis (SAS-MX Lipo-kit, Helena Biosciences, Gateshead, UK) was used to determine LDL purity. Samples were tested for endotoxin (Limulus amebocyte lysate test, BioWhittaker, Lonza, Visp, Switzerland) contamination that proved to be negative in all cases. LDL preparations were used within 48 h in the cell culture studies. Thiobarbituric-acid-reactive substance (TBARS) formation was assessed to exclude LDL oxidation in the sample preparations by a modified Ohkawa method [18,19]. Aggregated LDL (agLDL) was prepared by vortexing of the plasma purified LDL (1 mg/mL) to obtain 100 µg/mL agLDL as regularly performed in our group [9,10,11].

### 2.2. Scratch-Wound Assay

Double-sided scrape wounds were made on confluent hVSMC monolayers grown on 10 cm diameter plates, as previously described [9]. After wounding, the cells were washed with PBS and maintained in M199 migration medium (10% FCS) at 37 °C in a humidified atmosphere of 5% CO_2_ for 4 h time-periods. Cell migration and wound repair were controlled along the linear scratch using an inverted microscope (Leica DMIRE2) with a 10× lens magnification. After 4 h incubation, cells located in the wounded area or at the border of the wound were collected for gene and protein analysis and identified as migrating cells. In addition, subsets of monolayer hVSMCs located more than 500 mm from the wound border were harvested and stored following the same procedure and used as a “non-migrating” cell subset. Alternatively, 4 h after the wound was made, the cells were fixed with 4% paraformaldehyde for immunolabeling and confocal microscopy analysis.

### 2.3. Cell Adhesion Assay

Cell adhesion studies were performed as previously described [11]. Briefly, subconfluent cultures of hVSMCs were incubated with or without iC3b (100 nM), in the presence/absence of agLDL (100 µg/mL), for 24 h. Cells were then harvested and seeded on FBS-coated glass-bottom dishes in the presence or absence of iC3b and/or agLDL. At 1 and 3 h, cells were fixed with 4% paraformaldehyde for confocal microscopy analysis. Furthermore, attached cells were released by trypsination for determination of cell viability.

### 2.4. RNA Extraction and Real-Time PCR Analysis

Total RNA was extracted from hVSMCs by the RNeasy Mini Kit (Qiagen, Hilden, Germany, ref. 74104), according to the manufacturer’s instructions. RNA concentration was determined with a NanoDrop ND-1000 spectrophotometer (Thermo Fischer Scientific, Waltham, MA, USA) and purity was checked by the A260/A280 ratio.

Expression of motility-related genes was analyzed with the 96-well Human Target RT2 Profiler PCR Array from Qiagen (PAHS-128Z, Qiagen). This PCR array contains specific primers for 84 genes, 5 endogenous genes, and 7 controls. ACTB and GAPDH were used as endogenous controls. To analyze expression of individual genes, reverse transcription was performed with the High-Capacity cDNA Reverse Transcription Kit followed by TaqMan Real-Time PCR amplification (ThermoFisher Scientific, Waltham, MA, USA), according to the manufacturer’s instructions. Gene expression was analyzed using single assays (PXN: Hs01104424_m1; CTNNB1: Hs00355045_m1; FN1: Hs01549976_m1).

Samples were analyzed in duplicates and only genes with expression levels below 32 cycles were considered consistent. GAPDH (Hs02786624_g1) was used endogenously to normalize the expression levels by using the ΔCt method, according to the equation 2−(Ct [target]−Ct[endogenous]) [20]. All data were analyzed by SDS 2.4 and ExpressionSuite Software v1.1 (ThermoFisher Scientific).

### 2.5. In Silico Analysis

Genes involved in the modulation of actin cytoskeleton and gene/protein interaction networks were obtained by STRING (PubMed and Protein query) from the Cytoscape platform v3.10 (https://cytoscape.org/), San Diego, USA. Biological attributes were defined by gene ontology terms and signaling pathways by ShinyGO (https://bioinformatics.sdstate.edu/go/).

The Cytoscape platform was used to provide a basic set of features for data integration, analysis, and visualization that is maximized by apps for molecular profiling, new layouts, additional scripts, and different database connections. ShinyGO is an intuitive, graphical tool for enrichment analysis.

### 2.6. Western Blot Analysis

Total protein was extracted from hVSMCs with RIPA buffer (50 mM Tris HCl pH 8.0, 150 mM NaCl, 0.5% Triton X-100, 0.5% sodium deoxycholate, 0.1% SDS) [21] and Western blotting was performed as described [10] using the following primary antibodies: PXN (paxillin clone 5H11, 05-417, dilution 1/1000, Merck, Burlington, MA, USA), β-actin (Abcam Inc., Cambridge, MA, USA ab8226, dilution 1/5000). Total protein (Ponceau staining) was used as loading control. Detection of Western blot bands was performed by chemiluminescence technology (Supersignal, ThermoFisher, Rockford, IL, USA) and quantified with a ChemiDoc™ XRS system using Image Lab software v6.0.1 (Bio-Rad, Hercules, CA, USA).

### 2.7. Confocal Microscopy

Cells fixed with 4% paraformaldehyde, permeabilized (0.5% Tween-PBS), and blocked with 1% bovine serum albumin (BSA) were labeled for PXN and F-actin as previously described [9,10]. Signal detection was performed by using a specific anti-PXN antibody (clone 5H11 05-417-Millipore, dilution 1/50) with Alexa Fluor 488 or 633 as anti-mouse secondary antibody (Molecular Probes) and 633- or 488-phalloidine to visualize F-actin (Molecular Probes). Negative controls of primary and secondary antibody staining were run with each set of experiments. Cells were imaged using an HCX PL APO 63x/1.2 W Corr/0.17 CS objective on a Leica TCS SP2-AOBS inverted fluorescence microscope. Images were acquired at a resolution of 1024 × 1024 pixels in a spatial xyz mode (step size 0.1 mm) across a total of 20 optical sections and processed with the Leica Standard Software TCS-AOBS v2.7.7. Negative controls without primary antibody showed no detectable fluorescence labeling.

For colocalization analysis, the correlation between fluorescent signals was visualized in a two-dimensional cytofluorogram, with overlapping regions shown in yellow. Only the central cloud along the y–x axis of the cytofluorogram was selected. This is shown in yellow in the images.

The Pearson correlation (PC) coefficient indicates the degree of overlap in the color displays by describing the strength of the correlation between the distribution of the gray scale value in the first color channel (red, PXN signal) and the second channel (green, F-actin signal). The PC coefficient has values between +1 and −1. A value of +1 corresponds to total colocalization, while a value of −1 indicates no colocalization. Colocalization is recognized with a PC value greater than 0.5. Manders’ overlap coefficient (OA) indicates the overlap of fluorescent signals in both color channels and shows the extent of colocalization. This value ranges from 0 to 1; the closer it is to 1, the stronger the colocalization.

The colocalization rate (CR) is calculated by dividing the area of colocalizing fluorescent signals (colocalization area, CA) by the area of the image foreground (area foreground, AF) and the result is expressed as a percentage. The colocalization area (CA) specifies the area of colocalizing fluorescent signals in µm^2^, and the area of the image (AI) is the total area of the image in µm^2^. The area foreground (AF) is the area of the image with a fluorescent signal, and the area background (AB) is the area without a fluorescent signal.

### 2.8. Statistical Analysis

Results are expressed as mean ± standard error of the mean (SEM) except when indicated. Statistical differences between groups were calculated by the parametric paired Student’s *t*-test or the non-parametric Mann–Whitney and paired Wilcoxon tests. The statistical analysis was carried out using StatView 5.01 software (Abacus concepts, Piscataway, NJ, USA). *p* < 0.05 was considered statistically significant. * *p* < 0.05, ** *p* < 0.01, *** *p* < 0.001.

## 3. Results

### 3.1. Gene Profiling Related to Cell Migration of hVSMCs

A gene expression array comprising 84 genes related to cell motility (Profiler-PCR Array) was used to compare the expression profiles of migrating versus non-migrating human VSMCs (hVSMCs), as summarized in Figure S1.

**Figure 1:**
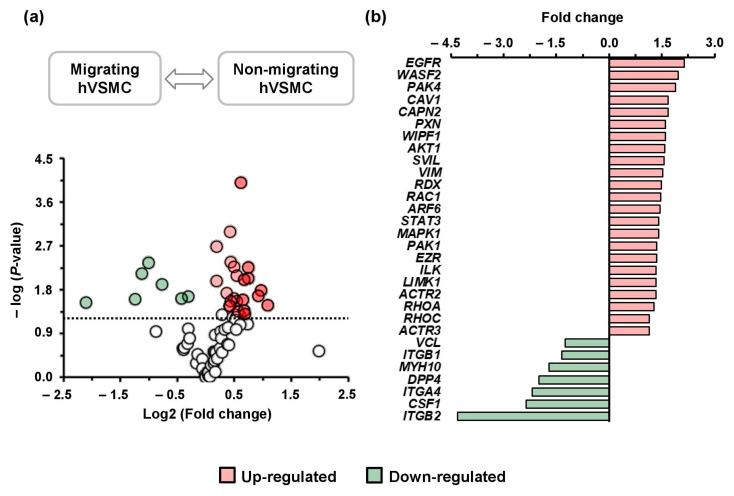
Gene expression profiling (**a**) Migrating and non-migrating hVSMCs (N = 3/group) analyzed by PCR array. Gene profiling is shown as volcano plot, *X*-axis represents log2 (fold change, migrating cells/non-migrating cells) and *Y*-axis represents -log10 (*p*-values). Significant changes (*p* > 1.3, −log10 (0.05)) are shown by red dots (upregulated genes) and green dots (downregulated genes). (**b**) The significant differential genes are shown in a bar plot according to fold change.

Over 90% of the genes included in the array were consistently detected in hVSMCs, with Ct values < 30. Among them, 30 genes (66% of the expressed genes) showed statistically significant changes in migrating cells 4 h after wounding, as illustrated in the volcano plot (Figure 1a). Of these, 23 genes were upregulated and 7 downregulated. The highest fold changes were observed for EGFR (2.13-fold) and ITGB2 (−4.32-fold) (Figure 1b and Table S1). These findings show that hVSMC migration is associated with marked transcriptional changes in adhesion- and cytoskeleton-related genes, guiding subsequent pathway analyses.

### 3.2. Gene Ontology (GO) Terms for the Differential Gene Pattern in Migrating hVSMCs

The 30 differentially expressed genes identified in migrating hVSMCs were analyzed using ShinyGO to determine associated biological processes (BP), molecular functions (MF), and cellular components (CC) based on Gene Ontology (GO) annotation terms (Figure S2). Enrichment analysis based on the top five functional categories (ranked by FDR values, fold enrichment, and number of genes) identified actin-filament-based process and cytoskeleton organization as the BPs with a higher fold enrichment (13.76 and 8.29, respectively). In addition, the most represented MFs were actin binding and protein kinase binding with fold enrichments of 13.61 and 9.94, respectively. The most represented subcellular locations were lamellipodia and focal adhesion, with fold enrichment values of 33.97 and 26.12, respectively (see Figure S2).

GO enrichment analysis revealed that migration-associated genes in hVSMCs are predominantly linked to actin cytoskeleton regulation and focal adhesion structures.

### 3.3. Differential Pattern of Genes Related to Actin Cytoskeleton in Migrating hVSMCs

In silico analysis was performed using the STRING PubMed database to identify genes involved in the regulation of the actin cytoskeleton. The search focused on the top 100 genes associated with VSMC migration, cytoskeleton organization, and focal adhesion. Thirteen genes were found to be common across all three search terms: PXN, AKT1, RHOA, VCL, CDC42, MYLK, PTK2, SRC, ROCK1, RAC2, CTNNB1, FN1, ACTB.

**Figure 2:**
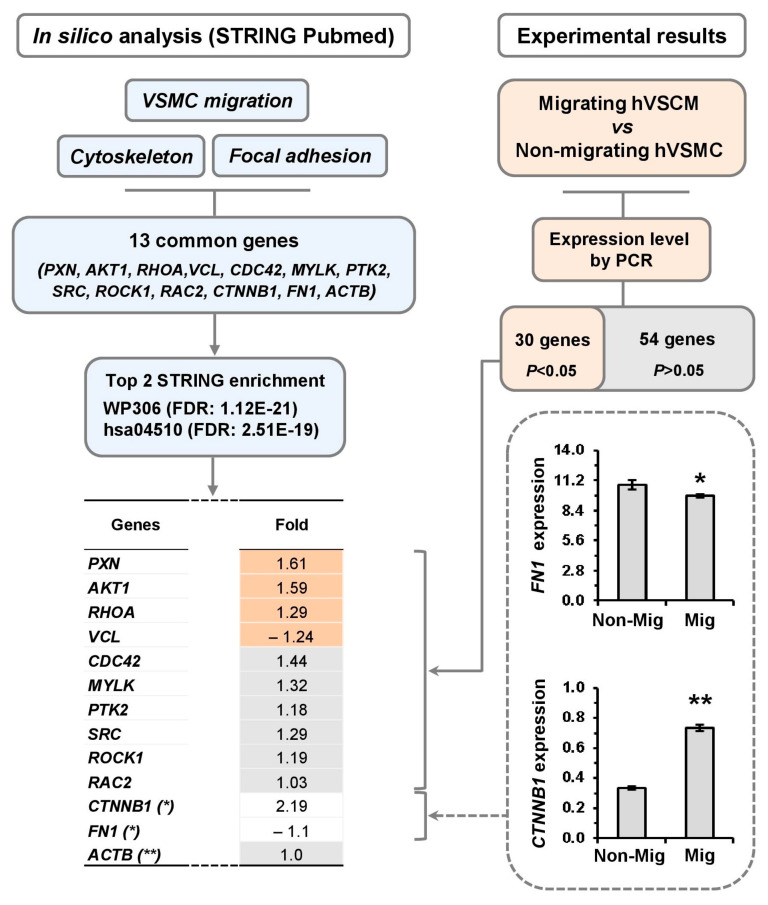
Identification of gene signatures in cytoskeleton remodeling. Genes obtained from STRING PubMed database in three categories (VSCM migration, cytoskeleton, focal adhesion) were analyzed by STRING enrichment tool and compared to the differential genes from the PCR array results, non-migrating (Non-Mig) vs. migrating (Mig). Gene expression levels of CTNNB1 and FN1 analyzed by single assay as extra PCR results. Bar plot represents mean  ±  SEM of three independent experiments (N = 3/group). * *p* < 0.05, ** *p* < 0.01.

Further in in silico validation using STRING’s enrichment analysis tool revealed that these 13 genes were also significantly enriched in other databases, including KEGG (hsa04510: focal adhesion) and WikiPathways (WP306: actin cytoskeleton), which emerged as the top two functional categories (FDR = 2.51 × 10^−19^ and 1.12 × 10^−21^, respectively) (Figure 2).

Ten of the thirteen genes identified through the in silico analysis were included in the Profiler-PCR Array. Among these, four genes (PXN, AKT1, RHOA, VCL) were differentially expressed in migrating hVSMCs. FN1 and CTNNB1, which were not included in the Profiler-PCR Array, were analyzed separately by RT-qPCR. FN1 was decreased, whereas CTNNB1 has increased expression during cell migration (Figure 2). These findings highlighted six key genes, PXN, AKT1, RHOA, VCL, CTNNB1, and FN1, linked to actin cytoskeleton remodeling and focal adhesion in migrating hVSMCs.

### 3.4. Signaling Pathway Linked to Cell Migration of hVSMCs

**Figure 3:**
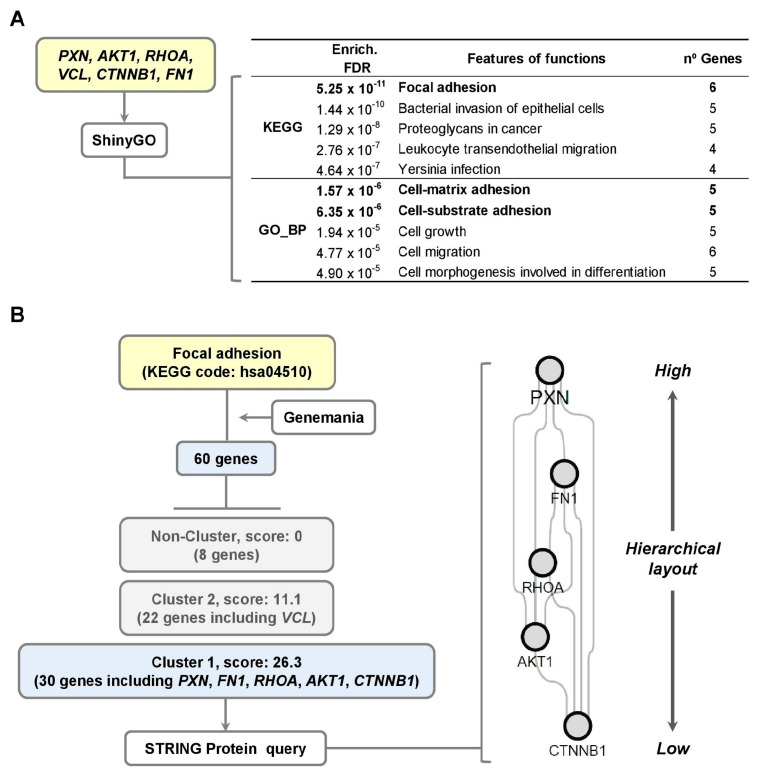
Regulation of cytoskeleton and molecular interaction in migrating hVSMCs. (**A**) Top 5 features of functions for KEGG pathways and biological process (GO_BP) related to PXN, AKT1, RHOA, VCL, CTNNB1, and FN1 obtained from ShinyGO database. (**B**) Identification of clusters by scoring method of genes included in Focal adhesion by Genemania. Protein interaction of the five proteins from Cluster 1 by STRING Protein query using hierarchical layout.

To investigate cytoskeletal regulation involving PXN, AKT1, RHOA, VCL, FN1, CTNNB1 and their molecular interactions in migrating hVSMCs, we performed an in silico analysis using ShinyGO (KEGG and biological process (BP)) and Cytoscape 3.10 (Genemania and STRING protein query) (Figure 3). Focal adhesion (KEGG pathway) was identified as the most significantly enriched signaling pathway (FDR: 5.25 × 10^−11^), including all six genes (see Figure 3A and Figure S3). Associated biological processes included cell–matrix adhesion and cell–substrate adhesion (Figure 3A).

GeneMANIA-based molecular interaction analysis revealed that the 60 genes involved in the focal adhesion pathway (KEGG pathway code: hsa04510) clustered into two groups: Cluster 1 (30 genes, score: 26.3) and Cluster 2 (22 genes, score: 11.1). PXN, FN1, RHOA, and AKT1 were included in Cluster 1, while CTNNB1 and VCL were part of Cluster 2 (Figure 3B).

Further STRING protein query analysis, visualized using a hierarchical layout, highlighted directional signaling within the focal adhesion network, with PXN at the highest hierarchical position and CTNNB1 at the lowest (See Figure 3B). These findings underscore focal adhesion as the key signaling pathway in migrating hVSMCs and support the selection of PXN as a central node for further analysis.

### 3.5. Effect of Atherogenic LDL on PXN Expression and Intracellular Localization

**Figure 4:**
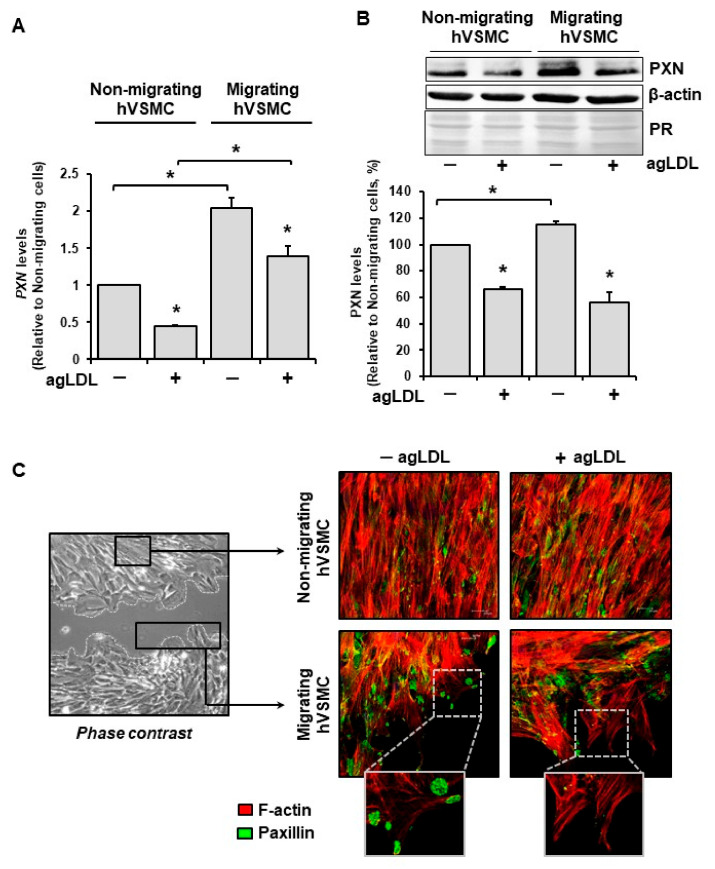
Gene and protein expression of PXN in hVSMCs stimulated with agLDL. (**A**) PXN expression by real-time PCR and (**B**) PXN concentrations by Western blot in migrating and non-migrating hVSMCs in presence or absence of 100 µg/mL agLDL (+agLDL hVSMCs or −agLDL hVSMCs). PR refers to Ponceau red for total protein staining. Bar plot represents mean ± SEM of three independent experiments (N = 3/group). * *p* < 0.05. (**C**) Migrating and non-migrating cells observed by phase-contrast microscopy and confocal microscopy. Signal detection and distribution of PXN and F-actin at the leading edge of the migrating and non-migrating hVSMCs with or without agLDL are shown. Notice the positive PXN (green) signal in podosome-like structures at the leading edge of −agLDL cells. Images are representative of 3 independent experiments.

PXN expression was evaluated in migrating hVSMCs when exposed to aggregated LDL (agLDL, 100 µg/mL, 24 h). Real-time PCR, Western blot, and confocal microscopy were used to assess PXN mRNA levels, protein expression, and subcellular localization, respectively (Figure 4).

PXN gene expression was upregulated in migrating cells entering the wounded area compared to non-migrating cells, both in the absence and presence of agLDL (–agLDL hVSMC and +agLDL hVSMC, respectively) (Figure 4A). At the protein level, PXN was upregulated in migrating hVSMCs under basal conditions (without agLDL), but this upregulation was not observed in the presence of agLDL (+agLDL hVSMC group) (Figure 4B). In addition, PXN mRNA and protein were downregulated by agLDL in non-migrating and migrating hVSMCs (see Figure 4A,B).

By confocal microscopy, PXN was found to be located in discrete, rounded structures at the leading edge of migrating hVSMCs, consistent with podosome-like structures (Figure 4, enlarged box). Fluorescence signals at the wound edge were less evident in cells exposed to agLDL (+agLDL) than in the absence of agLDL (−agLDL) (Figure 4C). Colocalization of PXN and F-actin showed a more consistent fluorescence signal concentrated at the cell edge in +agLDL hVSMCs than in −agLDL hVSMCs at 30 min (Figure S4). PXN fluorescence was significantly higher in the nuclear area than in the cytoplasm, and F-actin was detected throughout the cell, evidencing lamellipodia extensions after 60 min in the −agLDL hVSMCs. In addition, agLDL-induced cytoskeletal remodeling showed greater colocalization of PXN and F-actin in the endoplasmic region compared to the ectoplasmic region (see Figure S4).

In short, agLDL reduced PXN expression and altered its subcellular distribution in migrating hVSMCs, pointing to impaired cytoskeletal remodeling. We next explored potential molecular interactions linking PXN to cytoskeletal and adhesion-related proteins during cell migration.

### 3.6. PXN as a Target of iC3b-Integrin Signaling: In Silico Predictions and Confocal Analysis

**Figure 5:**
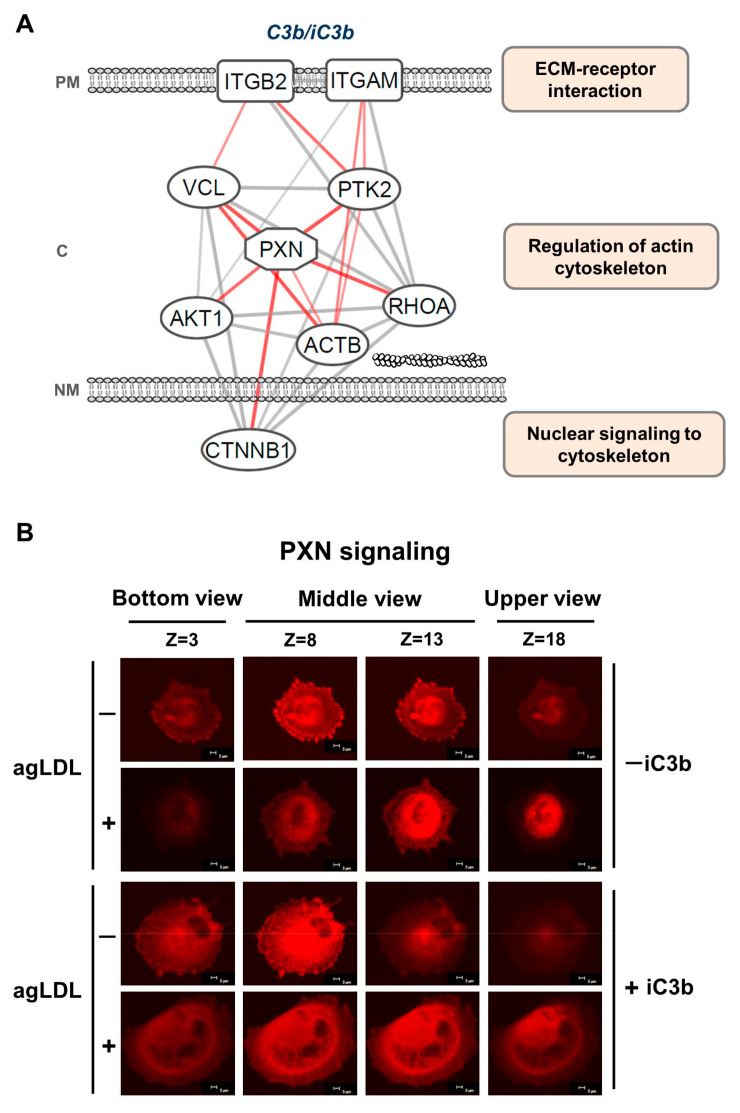
PXN regulation by immune signaling in lipid-loaded adherent hVSMCs. (**A**) Working model of molecular protein interactions involving PXN obtained through STRING Protein query. The network integrates experimentally relevant molecules with literature-supported associations, linking PXN to C3b/iC3b receptors (ITGB2/ITGAM) and to proteins involved in nuclear signaling, actin cytoskeleton regulation, and extracellular matrix (ECM)–receptor interactions. Gray lines represent protein interactions. Red lines indicate PXN-mediated connections between nuclear signaling, cytoskeleton, and extracellular matrix (EMC)–receptor interaction. PM: plasma membrane, C: cytoplasm, and NM: nuclear membrane. (**B**) Experimental validation of PXN intracellular distribution patterns by confocal microscopy in hVSMCs treated with or without iC3b (100 nM) in the absence/presence of agLDL (100 µg/mL). Images are representative of 3 independent experiments and were taken at intervals of 0.1 mm (20 slides) from the bottom (z = 3), middle (z = 8 and z = 13), and upper (z = 18) slides.

We performed an in silico STRING analysis to explore potential molecular interactions involving PXN. The analysis generated a working model (Figure 5A) linking PXN and the transcription factor CTNNB1, which connects nuclear signaling to the cytoskeleton; to cytoplasmic molecules involved in actin cytoskeleton regulation, including AKT1, ACTB, RHOA, VCL, and PTK2; and to plasma membrane receptors mediating extracellular matrix (ECM) interactions, such as the integrins ITGB2 and ITGAM (Figure 5A). These integrins also act as receptors for C3 complement activation products C3b/iC3b and are consistently expressed in hVSMCs [15]. To further investigate the potential effect of iC3b on PXN localization, we analyzed PXN distribution in hVSMCs using confocal microscopy.

PXN distribution in the cells was assessed by serial z-stack analysis across 20 optical sections, focusing on defined cellular depths (bottom: z = 3; middle: z = 8–13; top: z = 18) in hVSMCs treated with or without iC3b in the absence/presence of agLDL (Figure 5B). In control cells, PXN signal was predominantly localized at the bottom (z = 3) and low middle (z = 8) planes, with a diffuse distribution throughout the cell, and with decreasing intensity from z = 13 to the top (z = 18), consistent with PXN enrichment at anchoring sites. In +agLDL hVSMCs, PXN fluorescence shifted toward upper cell sections, showing higher intensity at z = 13 and z = 18 and displaying a more concentrated perinuclear/endoplasmic pattern.

In the presence of iC3b, PXN exhibited a stronger signal and broader distribution across all z-planes in lipid-loaded cells (+agLDL hVSMCs, +iC3b), whereas, in the absence of agLDL (−agLDL group, +iC3b), PXN signal remained mainly localized at the cell bottom (from z = 3 up to z = 8; see Figure 5B). These patterns indicate a displacement of PXN from the anchorage sites to other cellular compartments in lipid-loaded hVSMCs.

Additionally, cell spreading area, calculated from the projected image obtained by merging all 20 z-stack slices, was significantly greater in iC3b-treated cells, irrespective of agLDL exposure (Supplementary Figure S5). These results support the hypothesis, generated from the STRING network in panel A, that αMβ2-mediated signaling modulates PXN redistribution in lipid-loaded hVSMCs.

**Figure 6:**
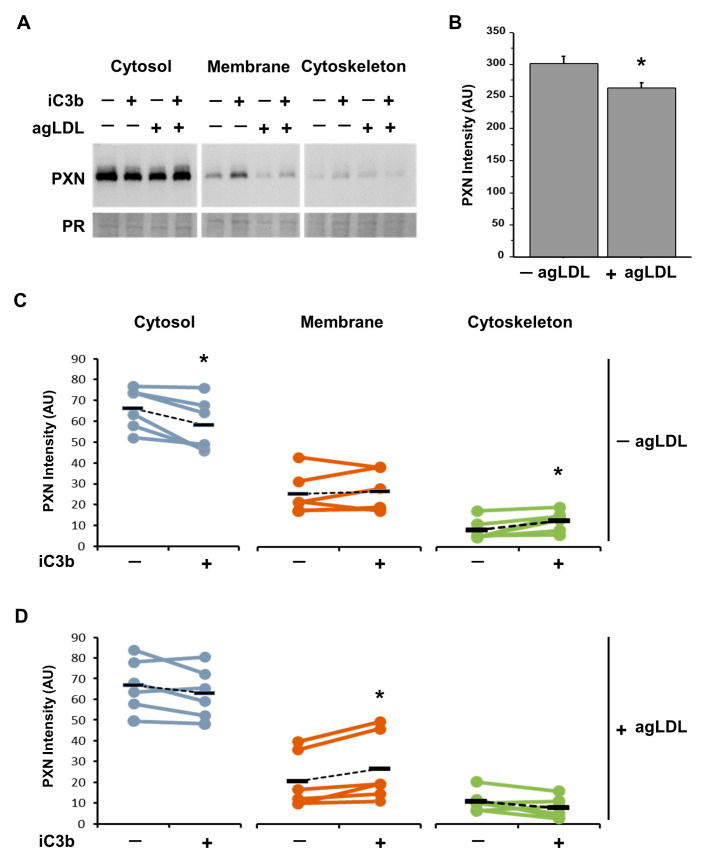
Cellular fractions of PXN protein in lipid-loaded adherent hVSMCs by adding iC3b PXN expression in cytosol, membrane, and cytoskeleton fractions, with or without LDL (100ug/mL) in the presence or not of iC3b (100 nM). (**A**) Representative image of Western blot (N = 6/group). PR: Ponceau red, loading control. (**B**) PXN expression considering the sum of all fractions for −agLDL hVSMCs vs. +agLDL hVSMCs. Bar plot represents mean ± SEM of three independent experiments (N = 3/group). * *p* < 0.05. (**C**) PXN in each subcellular fraction calculated as percentage of band intensity related to total intensity in absence of agLDL and (**D**) presence of agLDL. * *p* < 0.05.

Western blot analysis of cell subfractions sequentially extracted from hVSMCs revealed >2-fold higher PXN intensity in the cytosol than in membrane and cytoskeleton samples (Figure 6A). The presence of agLDL in adherent hVSMCs showed lower protein expression of PXN compared with −agLDL hVSMCs (Figure 6B).

In the absence of agLDL, iC3b induced a significant reduction of PXN in the cytosol fraction and a significant increase in the cytoskeletal fraction, while PXN in the membrane fraction was not modified (Figure 6C). On the contrary, in +agLDL hVSMCs, the effect of iC3b on PXN did not show significant changes in the cytosol and cytoskeletal fractions, but PXN was increased in the membrane fraction (Figure 6D).

### 3.7. iC3b Modulates the Cytoskeleton Remodeling of PXN in Lipid-Loaded Adherent hVSMCs

**Figure 7:**
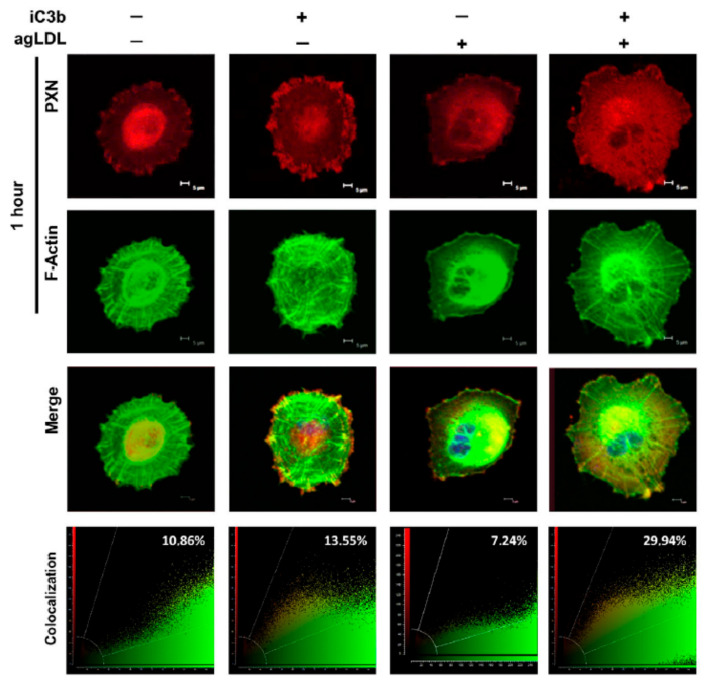
Cytoskeleton remodeling of PXN and F-actin by iC3b in lipid-loaded adherent hVSMCs. Fluorescent signal of PXN, F-actin, and their colocalization determined by confocal microscopy analysis in presence or absence of agLDL (100 ug/mL) (−agLDL and +agLDL hVSMCs) for 1 h and/or iC3b (100 nM). Images are representative of 3 independent experiments and were taken at intervals of 0.1mm (20 slides).

Regulation of actin cytoskeleton by iC3b in agLDL-treated hVSMCs was evidenced by the colocalization of PXN and F-actin, as shown in Figure 7. In the absence of iC3b, the PXN–F-actin colocalization was lower in agLDL-treated (+agLDL) compared to untreated (−agLDL) cells (7.24% and 10.86%, respectively). In these conditions, PXN localization was primarily observed in the endoplasmic region (+agLDL) and the nuclear area (−agLDL).

Upon iC3b treatment, lipid-loaded hVSMCs (+iC3b, +agLDL) showed a marked increase in the colocalization between PXN and F-actin compared to untreated (−agLDL) hVSMCs (19.68% vs. 9.38%). In addition, the subcellular distribution pattern shifted, with high colocalization in the nuclear region in cells treated with agLDL but without C3b and a peripheral localization pattern in cells treated with both agLDL and iC3b, as shown in Figure 7. Statistical analysis for colocalization rates between F-actin and PXN evidenced a significant increase in the iC3b-treated hVSMCs, unrelated to the exposure to agLDL (+agLDL/iC3b vs. +agLDL: CR: 29.9% vs. 7.2%, *p* = 0.021; −agLDL/iC3b vs. −agLDL: CR: 13.5% vs. 10.9, *p* = 0.028). No statistical differences were identified in the CR levels between lipid-loaded and control hVSMCs in the absence of iC3b.

## 4. Discussion

In this study, we used a targeted platform to investigate gene expression patterns related to cell migration, cytoskeletal remodeling, and cell adhesion hVSMCs. We first analyzed migration-associated gene networks, then assessed how lipid loading alters focal adhesion organization, and finally examined how C3 complement activity (iC3b) modulates cell–matrix adhesion under lipid-loaded and non-lipid-loaded conditions. This sequential approach allowed us to connect molecular findings with functional changes in adhesion and cytoskeletal dynamics, providing a rationale for integrating these experimental components into a single, hypothesis-generating, pathway-focused framework.

Regulation of the actin cytoskeleton is a key biological process involved in cellular adaptation [21] and plays a role in various pathophysiological conditions, including atherosclerosis [22]. The migration of VSMCs is tightly associated with cytoskeleton remodeling and plays a critical role in the maintenance of atherosclerotic plaque stability [23]. However, the gene regulatory networks governing cytoskeletal dynamics in the context of VSMC migration are not yet fully elucidated. Here, we have performed a comprehensive in silico analysis from a differential gene expression analysis of migrating hVSMCs. Real-time PCR profiling identified 30 genes with significant expression changes in migrating versus non-migrating hVSMCs. Among these, six genes—PXN, AKT1, RHOA, VCL, CTNNB1, and FN1—were associated with actin cytoskeleton regulation/function based on STRING pathway analysis. These findings suggest that approximately 20% of the differentially expressed genes in migrating hVSMCs may contribute to cytoskeletal remodeling.

To refine our focus on the genes most strongly associated with cytoskeletal regulation, we subjected these six candidates to hierarchical analysis using the STRING algorithm. PXN emerged as the top-ranked gene, suggesting a central regulatory role in cytoskeletal organization during VSMC migration.

PXN, a cytoskeleton-associated protein, functions as a key mediator in the transduction of extracellular signals into intracellular responses, regulating various cellular processes, including cell migration [24]. In our study, lipid-loaded hVSMCs consistently showed reduced PXN expression at the leading edge of migrating cells compared to non-migrating lipid-loaded hVSMCs. Confocal imaging revealed that PXN partially colocalized with F-actin in discrete, rounded structures at the leading edge, consistent with podosome-like structures described in migrating VSMCs and known to be involved in matrix interaction and turnover.

Thus, these findings indicate that lipid loading, as is the case in atherosclerotic lipid-rich plaques, not only impairs the migratory capacity of hVSMCs, as we had previously reported [9], but also alters the spatial distribution of PXN in the lamellipodia region during reduced cell migration.

Specifically, here we demonstrated for the first time that agLDL orchestrates the downregulation of PXN in cell migration of lipid-loaded hVSMCs. Supporting our findings, a previous study on PXN knockdown in hVSMCs showed a decrease in cell migration that was reversed by inducing high levels of PXN [15]. Thus, these common findings supported our effort to perform further studies to investigate the role of PXN in the dynamic activity of the cytoskeleton within migrating lipid-laden hVSMCs.

In our studies, focal adhesion was the predicted signaling pathway related to PXN’s effects on the cytoskeleton. By confocal analysis, PXN showed a differential colocalization with F-actin in the endoplasmic area in lipid-loaded hVSMCs compared with control non-loaded hVSMCs.

Lipid-loaded hVSMCs by agLDL and lipid-induced chronic inflammation in the activated arterial wall are widely recognized in atherosclerosis processes [23]. The complement system has been related to vascular remodeling [25] and atherosclerosis [26]. Interestingly, our in silico analysis based on STRING revealed that PXN is involved in the signaling pathway of extracellular matrix (ECM)–receptors (integrins (αMβ2): ITGAM (CD11b) and ITGB2 (CD18)) and related to the C3 complement system [11]. Furthermore, in our previous study, our group found by mass spectrometry and differential proteomics that the ECM of atherosclerotic human aortic arteries is enriched in various active components of the C3 complement system compared to atherosclerosis-free aortic segments [11]. The C3 complement and its C3-activated fragment (C3b/iC3b) had been previously found in atherosclerotic human arteries [27,28]. Thus, it is plausible that the C3 proteolytic product (iC3b) is part of the circulating inflammatory response and its presence in vascular tissue may exacerbate the cytoskeletal remodeling in atherosclerosis. However, although there is a large body of literature linking cell adhesion to the cytoskeleton, the effect of atherogenic stimuli such as agLDL and iC3b on the regulation of the actin cytoskeleton of hVSMCs was unknown.

In this context, we have demonstrated that exogenous iC3b signaling is a key stimulus for cytoskeletal remodeling in lipid-loaded hVSMCs. Our in silico analysis suggests that the C3 product iC3b induces a distinct PXN distribution pattern during cell adhesion mediated by activation of the αMβ2 integrin. Interestingly, αMβ2 activation was shown to be necessary for paxillin phosphorylation on tyrosine, a key feature for cell migration [29]. Our findings warrant further dedicated studies to dissect the mechanistic signaling processes underlying the ITGB2/ITGAM–paxillin interaction in hVSMCs, including targeted silencing approaches, to clarify its role under different pathology-mimicking conditions. In line with these observations, our quantitative analysis of cell spreading area showed significantly higher values in iC3b-treated cells, independently of agLDL exposure, further supporting the role of iC3b in modulating hVSMC cytoskeletal organization.

Confocal microscopy revealed that, in +iC3b-lipid-loaded hVSMCs, PXN was distributed throughout the cell from the bottom to top, in contrast to the more localized perinuclear distribution in the upper cellular sections, observed in lipid-loaded hVSMCs not treated with iC3b.

In these lipid-loaded hVSMCs treated with iC3b, the colocalization of PXN with F-actin was more than 15-fold higher than in the lipid-loaded hVSMCs not treated with iC3b. In addition, subcellular fractionation analysis did not show significant changes in cytosolic PXN levels in the high-colocalization group, suggesting that the differential pattern in cell remodeling is given by cell distribution of PXN associated with regulation of the actin cytoskeleton rather than a regulated synthesis of PXN protein.

Thus, the atherogenic stimulus induced by agLDL, together with inflammatory signaling mediated by the iC3b complement appears to activate a relatively unexplored downstream pathway involved in the regulation of the actin cytoskeleton. Further studies are required to fully elucidate the underlying mechanisms.

Although our current findings are limited to in vitro models, the PXN–iC3b pathway could represent a potential therapeutic target for modulating VSMC behavior and stabilizing atherosclerotic plaques in vivo. Evidence that C3 deficiency can exacerbate plaque development in mouse models underscores the complex role of the complement in vascular homeostasis [13], while recent studies highlight paxillin as a critical mediator of VSMC cytoskeletal remodeling and migration through its interaction with the kinase Fyn (reviewed in [30]). Together, these data support the plausibility of targeting the iC3b–PXN axis for vascular remodeling interventions—a strategy that warrants dedicated experimental validation.

## 5. Conclusions

In conclusion, our findings show for first time a relationship between iC3b-derived molecular signaling and reorganization of the cytoskeleton associated with PXN distribution in lipid-loaded hVSMCs. This highlights a previously underexplored downstream signaling pathway extending beyond iC3b’s well-established roles in inflammation and immunity. These results suggest that iC3b-induced modulation of PXN and the actin cytoskeleton may represent novel contributors to vascular remodeling and the progression of atherosclerotic lesions.

## Data Availability

The data that support the findings of this study are available from the corresponding author upon reasonable request.

## Supplementary Materials

The following supporting information can be downloaded at: https://www.mdpi.com/article/10.3390/cells14161245/s1, Figure S1: Schematic diagram representing the study design; Figure S2: Biological attributes related to cell migration in hVSMC; Figure S3: Focal adhesion pathway (KEGG code: hsa04510); Figure S4: Localization pattern of PXN in adherent hVSMC; Figure S5: Cell spreading induced by iC3b. Table S1: Differential gene profiling in migrating hVSMC.
