# Supplementary material for: Gene Expression Pattern Associated with Cytoskeletal Remodeling in Lipid-Loaded Human Vascular Smooth Muscle Cells: Crosstalk Between C3 Complement and the Focal Adhesion Protein Paxillin
Source: Cells. 2025 Aug 12;14(16):1245. doi: 10.3390/cells14161245 (PMC12384099; doi:10.3390/cells14161245)
Supplement: Supplementary file 1 [file cells-14-01245-s001.zip › cells-3754311-supplementary.pdf]

cells

MDPI

Supplementary Materials

Gene Expression Pattern Associated with Cytoskeletal Remodeling in Lipid-Loaded Human Vascular Smooth Muscle Cells: Crosstalk Between C3 Complement and the Focal Adhesion Protein Paxillin

Maisa Garcia-Arguinzonis [1,†], Rafael Escate [1,2,†], Roberta Lugano [3], Esther Peña [1], Maria Borrell-Pages [1,2], Lina Badimon [1,2,4] and Teresa Padro [1,2,*]

[1] Institut Recerca Sant Pau (IR-Sant Pau), 08041 Barcelona, Spain; mgarciaar@santpau.cat (M.G.-A.); rescate@santpau.cat (R.E.); epena@santpau.cat (E.P.); mborrellpa@santpau.cat (M.B.-P.); lbadimon@ficsi.org (L.B.)

[2] Centro de Investigación Biomédica en Red Cardiovascular (CIBERCV), Instituto de Salud Carlos III, 28029 Madrid, Spain

[3] Candiolo Cancer Institute, Fondazione del Piemonte per l'Oncologia (FPO-IRCCS), Candiolo, 10060 Torino, Italy; roberta.lugano@ircc.it

[4] Medical School, Universitat de Vic–UCC, Cardiovascular Research Foundation for Health Prevention and Innovation (FICSI), 08017 Barcelona, Spain

* Correspondence: tpadro@santpau.cat; Tel.: +34-935565886

† These authors contributed equally to this work.

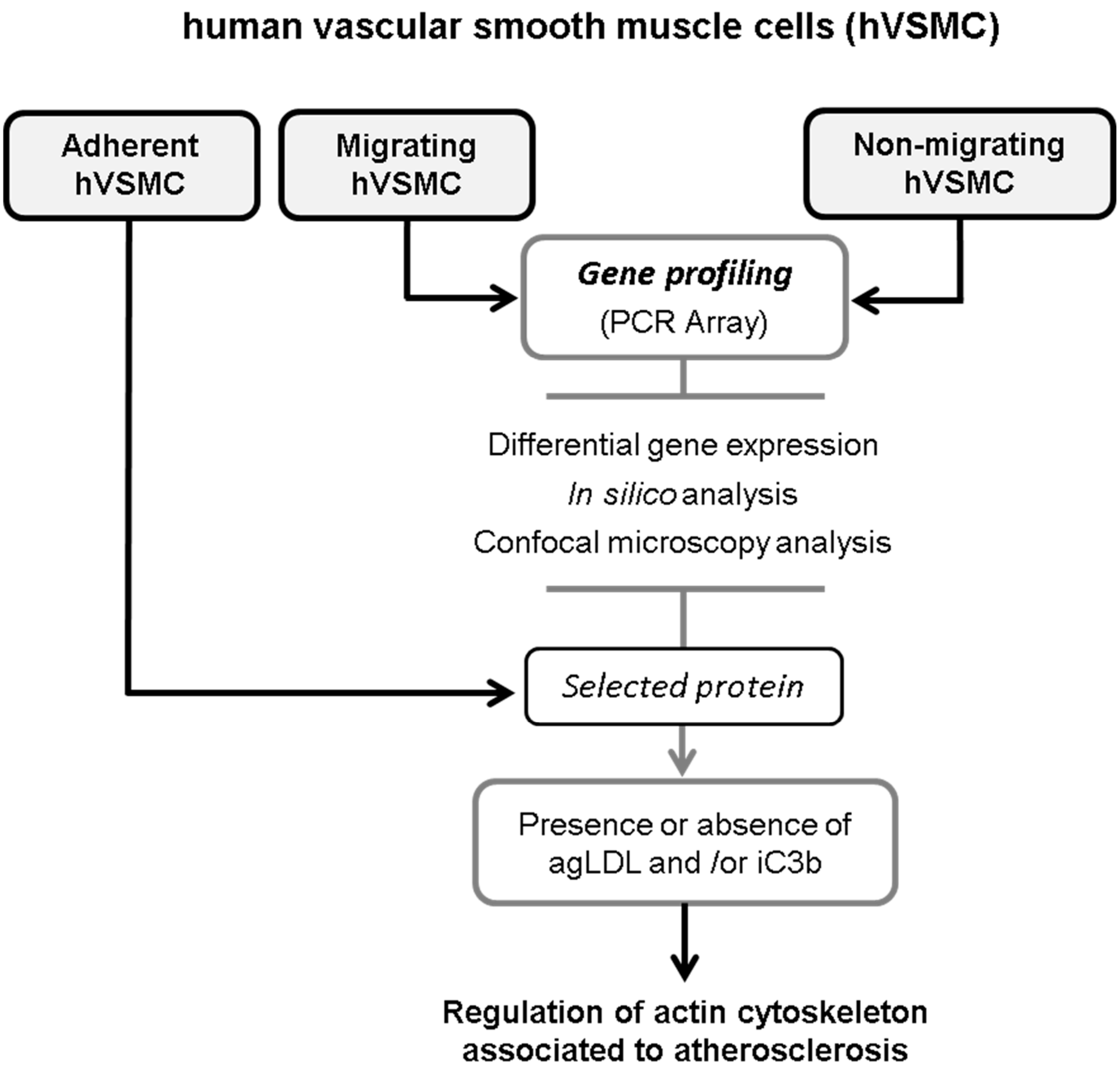

Figure S1. Schematic diagram representing the study design. Cell migration and adherent hVSMC exposed or not to atherogenic stimulus (agLDL and iC3b).

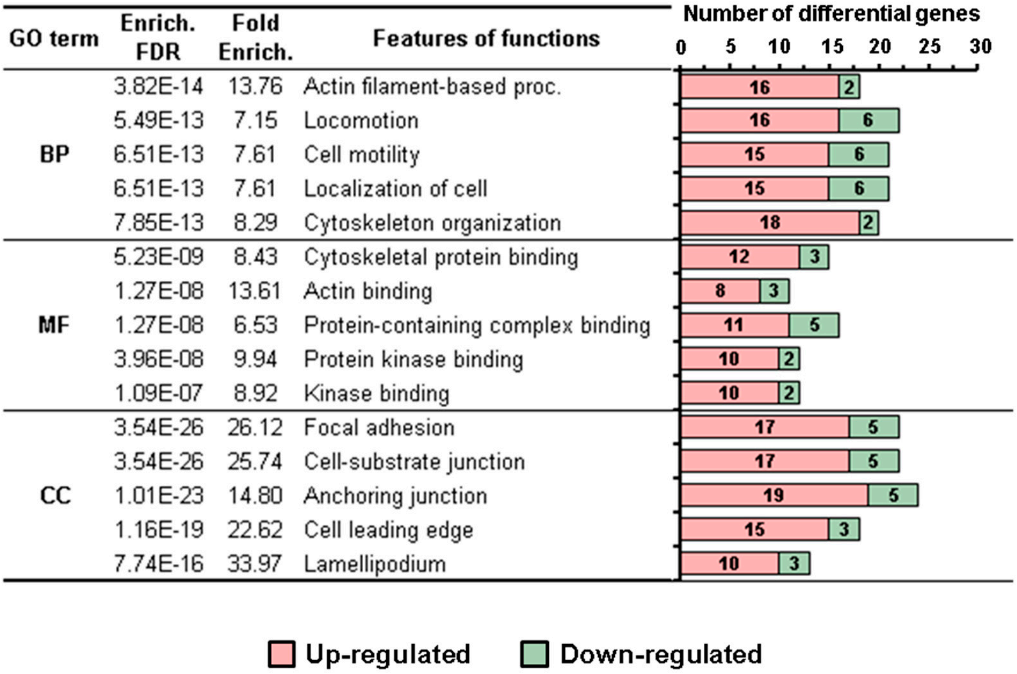

Figure S2. Biological attributes related to cell migration in hVSMC. Gene ontology enrichment analysis was performed by ShinyGO to the differential gene expression. Top 5 features of functions are attributed to Gene ontology (GO) terms for biological process (BP), molecular function (MF), cellular component (CC). Bar plot shows the number of differential genes involved (up-regulated and down-regulated). Enrich. FDR: Enrichment FDR, Fold enrich: Fold enrichment, FDR: false discovery rate.

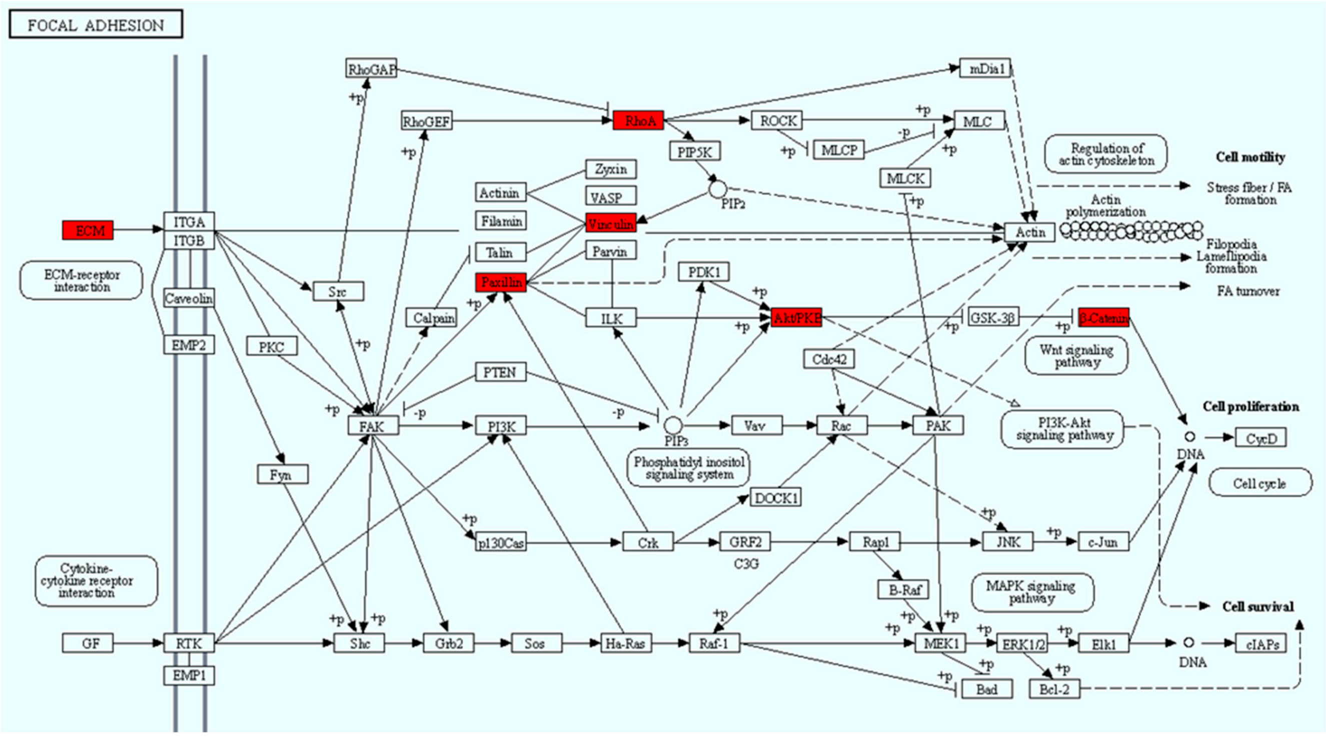

Figure S3. Focal adhesion pathway (KEGG code: hsa04510). Molecules in red indicate PXN (Paxillin), AKT1 (Akt/PKE), RHOA (RhoA), VCL (Vincullin), CTNNB1 (β-Catenin) and FN1 (ECM).

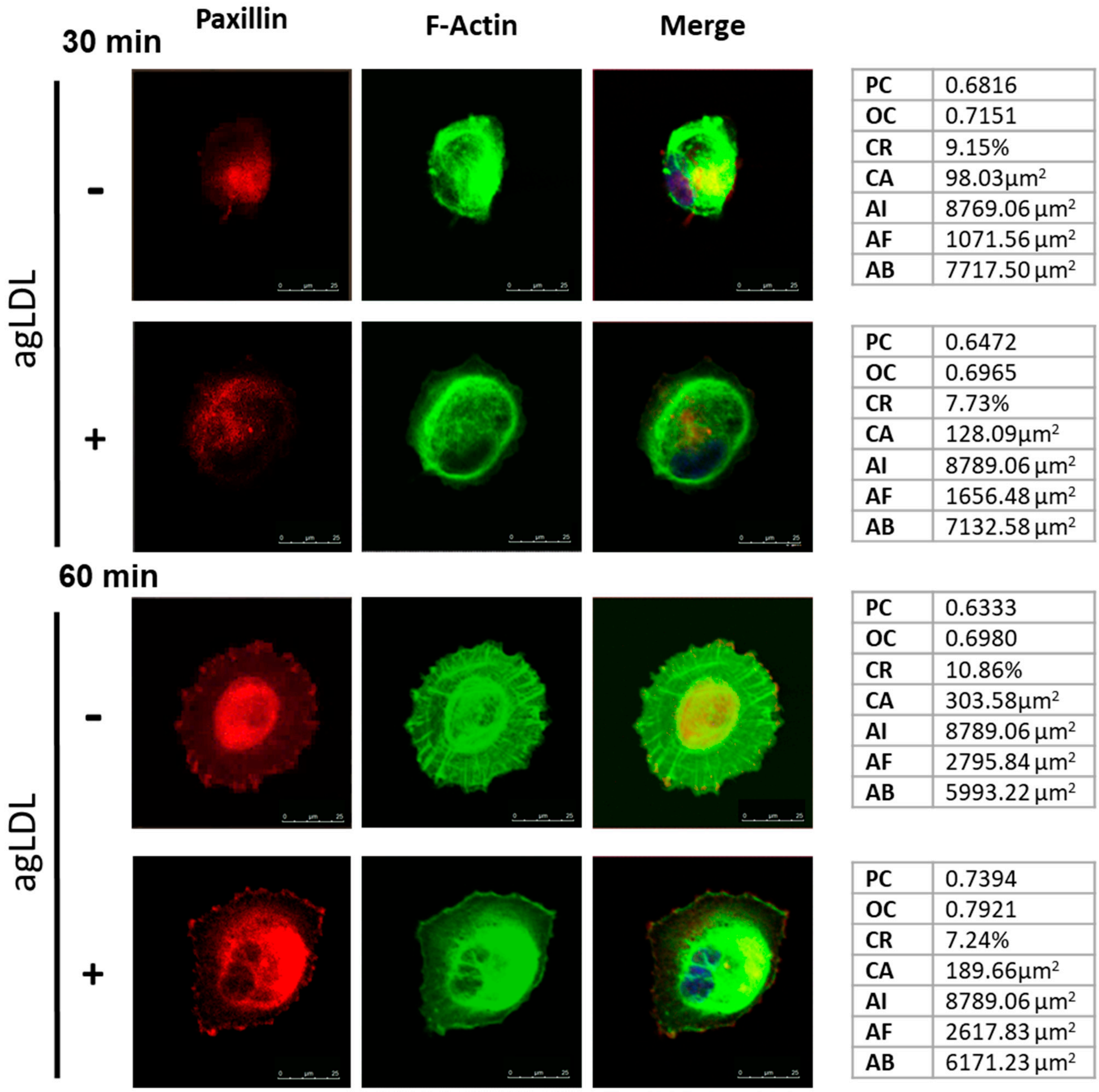

Figure S4. Localization pattern of PXN in adherent hVSMC. Confocal microscopy analysis of PXN and F-actin in hVSMC with or without agLDL (-agLDL hVSMC *vs* +agLDL hVSMC) for 30- and 60-min. Images were taken at intervals of 0.1 mm (20 slides) and are representative of 3 independent experiments. Colocalization of PXN and F-actin is shown, Pearson's correlation coefficient (PC) was used to describe the correlation between the intensity distributions of the two channels, colocalization is accepted with PC>0.5. Overlap coefficient (OC) quantified the proportion of overlapping signals ranging from 0 to 1. Colocalization rate is calculated as the area of colocalizing fluorescent signals (colocalization area, CA) /the area of the image foreground (AF) ×100. CA specifies the area of colocalizing fluorescent signals in µm², the area of the image (AI) is the total area of the image in µm², AF is the area of the image with a fluorescent signal, and the area background (AB) is the area without a fluorescent signal. Bar = 25µm.

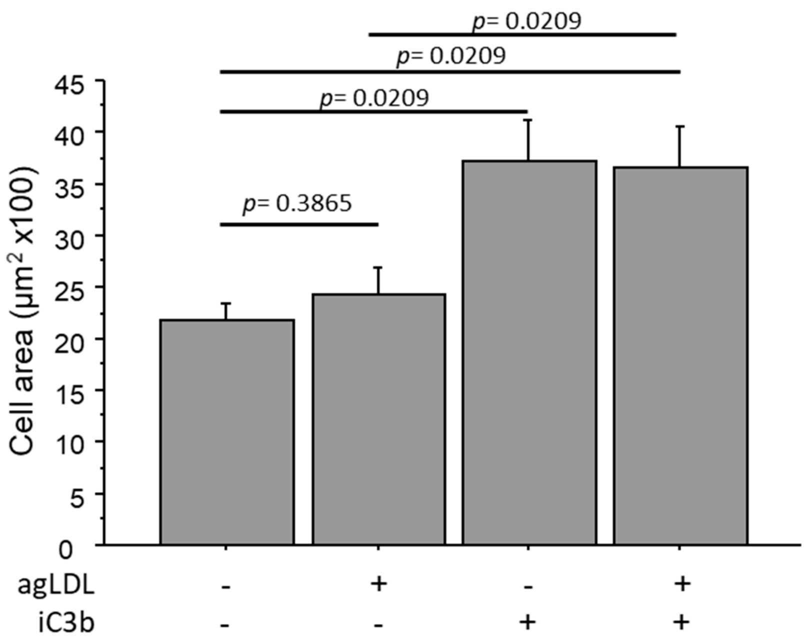

Figure S5. Cell spreading induced by iC3b. Cell spreading analysis was done measuring the projected confocal microscopy image cell area from images obtained by of hVSMC adhering for 1 hour, treated with or without agLDL in the presence or absence of iC3b. The area covered by the cell was calculated using Image J Fiji software, setting the scale for every cell. Significance has calculated with Mann-Whitney (n = 4) and Kruskal-Wallis (p = 0.0124) statistics.

Table S1. Differential gene profiling in migrating hVSMC.

nº	Genes	Fold	*p*-value	nº	Genes	Fold
1	***EGFR***	2.13	*	31	***SH3PXD2A***	0.54
2	***WASF2***	1.96	*	32	***EGF***	0.63
3	***PAK4***	1.89	*	33	***MYLK***	0.76
4	***CAV1***	1.68	**	34	***MMP14***	0.76
5	***CAPN2***	1.68	**	35	***BAIAP2***	0.77
6	***PXN***	1.61	*	36	***ITGB3***	0.80
7	***WIPF1***	1.60	*	37	***FAP***	0.81
8	***AKT1***	1.59	**	38	***MMP2***	0.82
9	***SVIL***	1.57	*	39	***MYL9***	0.89
10	***VIM***	1.53	***	40	***PLAUR***	0.91
11	***RDX***	1.48	*	41	***ACTN3***	0.94
12	***RAC1***	1.46	**	42	***MSN***	0.96
13	***ARF6***	1.45	*	43	***TLN1***	0.96
14	***STAT3***	1.41	*	44	***TIMP2***	0.99
15	***MAPK1***	1.41	**	45	***RHO***	0.99
16	***PAK1***	1.35	*	46	***ACTN1***	1.00
17	***EZR***	1.35	**	47	***PFN1***	1.21
18	***ILK***	1.34	**	48	***WASF1***	1.43
19	***LIMK1***	1.34	*	49	***PRKCA***	1.52
20	***ACTR2***	1.33	*	50	***RHOB***	1.39

21	***RHOA***	1.29	*
22	***RHOC***	1.14	**
23	***ACTR3***	1.14	*
24	***VCL***	-1.24	*
25	***ITGB1***	-1.35	*
26	***MYH10***	-1.70	*
27	***DPP4***	-2.00	**
28	***ITGA4***	-2.19	**
29	***CSF1***	-2.36	*
30	***ITGB2***	-4.32	*

51	***CDC42***	1.44
52	***CRK***	1.43
53	***HGF***	3.15
54	***MET***	1.66
55	***ARHGEF7***	1.52
56	***FGF2***	1.02
57	***RAC2***	1.03
58	***DIAPH1***	1.03
59	***PLD1***	1.03
60	***ENAH***	1.04
61	***PLCG1***	1.05
62	***VASP***	1.06
63	***MMP9***	1.07
64	***PTK2B***	1.08
65	***PTPN1***	1.10
66	***ACTN4***	1.10
67	***TGFB1***	1.10
68	***CAPN1***	1.11
69	***MYH9***	1.11
70	***VEGFA***	1.12
71	***CFL1***	1.12
72	***CTTN***	1.13
73	***BCAR1***	1.15
74	***PTK2***	1.18
75	***ROCK1***	1.19
76	***RASA1***	1.20
77	***WASL***	1.22
78	***IGF1R***	1.26
79	***SRC***	1.29
80	***PIK3CA***	1.30
81	***PTEN***	1.32
82	***IGF1***	1.43
83	***ARHGDIA***	1.45
84	***RND3***	3.97

Differential gene expression quantified by real-time PCR in migrating hVSMC compared with non-migrating hVSMC (Fold change). Genes in red: up-regulated. Genes in green: down-regulated. *$p < 0.05$, **$p < 0.01$, ***$p < 0.001$. Genes in bold: $p > 0.05$).
